# Aberrant ribonucleotide incorporation and multiple deletions in mitochondrial DNA of the murine MPV17 disease model

**DOI:** 10.1093/nar/gkx1009

**Published:** 2017-11-02

**Authors:** Chloe F. Moss, Ilaria Dalla Rosa, Lilian E. Hunt, Takehiro Yasukawa, Robert Young, Aleck W. E. Jones, Kaalak Reddy, Radha Desai, Sam Virtue, Greg Elgar, Peter Voshol, Martin S. Taylor, Ian J. Holt, Martin A. M. Reijns, Antonella Spinazzola

**Affiliations:** 1 MRC Laboratory, Mill Hill, London NW7 1AA, UK; 2 Department of Clinical Neurosciences, Institute of Neurology, Royal Free Campus, University College London NW3 2PF, UK; 3 Advanced Sequencing Facility, Francis Crick Institute, London NW1 1AT, UK; 4 MRC Mitochondrial Biology Unit, Cambridge CB1 9SY, UK; 5 MRC Human Genetics Unit, MRC Institute of Genetics and Molecular Medicine, The University of Edinburgh, Edinburgh EH4 2XU, UK; 6 MRC Metabolic Diseases Unit, Wellcome Trust-MRC Institute of Metabolic Science, Cambridge CB2 0QQ, UK; 7 Biodonostia Health Research Institute, 20014 San Sebastián, Spain and IKERBASQUE, Basque Foundation for Science, 48013 Bilbao, Spain; 8 MRC Centre for Neuromuscular Diseases, UCL Institute of Neurology and National Hospital for Neurology and Neurosurgery, Queen Square, London WC1N 3BG, UK

## Abstract

All DNA polymerases misincorporate ribonucleotides despite their preference for deoxyribonucleotides, and analysis of cultured cells indicates that mammalian mitochondrial DNA (mtDNA) tolerates such replication errors. However, it is not clear to what extent misincorporation occurs in tissues, or whether this plays a role in human disease. Here, we show that mtDNA of solid tissues contains many more embedded ribonucleotides than that of cultured cells, consistent with the high ratio of ribonucleotide to deoxynucleotide triphosphates in tissues, and that riboadenosines account for three-quarters of them. The pattern of embedded ribonucleotides changes in a mouse model of Mpv17 deficiency, which displays a marked increase in rGMPs in mtDNA. However, while the mitochondrial dGTP is low in the Mpv17^−/−^ liver, the brain shows no change in the overall dGTP pool, leading us to suggest that Mpv17 determines the local concentration or quality of dGTP. Embedded rGMPs are expected to distort the mtDNA and impede its replication, and elevated rGMP incorporation is associated with early-onset mtDNA depletion in liver and late-onset multiple deletions in brain of Mpv17^−/−^ mice. These findings suggest aberrant ribonucleotide incorporation is a primary mtDNA abnormality that can result in pathology.

## INTRODUCTION

Mammalian mitochondrial DNA (mtDNA) is a small covalently closed circular molecule of ∼16 kb, encoding 13 essential components of the oxidative phosphorylation system (OXPHOS). OXPHOS provides the bulk of the cell's energy in the form of ATP, thus a reduction in the amount (depletion) or quality of the mtDNA (ranging from point mutations to multiple deletions) can cause an energy crisis and human pathologies (1). A group of mtDNA disorders result from defects in factors that alter deoxynucleotide triphosphate (dNTP) homeostasis (2–4), one of which is MPV17, a mitochondrial inner membrane protein whose loss of function causes a tissue-specific decrease of dGTP and dTTP, which is associated with mtDNA depletion (5,6).

A peculiar feature of mammalian mtDNA is the high number of embedded ribonucleotides in comparison to nuclear DNA (7–9), which are scattered throughout both strands of the mtDNA (7,10). Although an early study detected no bias among the four ribonucleotides (11), more recently rCMP and rGMP were found to be disproportionately high in HeLa cell mtDNA, whereas rAMPs and rGMPs were the most abundant in a human fibroblast cell line (12). Why the embedded ribonucleotides are better tolerated by mtDNA than nuclear DNA is not completely understood, although, possible explanations are the small size of the mitochondrial genome, the slow rate of mtDNA synthesis (13) and the bacteriophage-like enzymes involved in its replication (PEO1, POLG and POLRM) (14). Moreover, several outstanding questions remain: (i) What is the upper limit for ribonucleotides in mammalian mitochondrial DNA? (ii) Does this threshold differ among the four bases? (iii) What is the variation in rNMPs among different cell and tissue types? (iv) Do incorporated ribonucleotides play a role in mtDNA diseases?

To address these questions we employed emRiboSeq (15), which makes use of type 2 RNase H cleavage at embedded ribonucleotides in isolated genomic DNA. We establish that ribonucleotide incorporation in mtDNA is much higher in solid tissues than in proliferating cells, with rAMPs representing over three-quarters of the total, from which we infer ATP to be the major source of incorporated ribonucleotides of mammalian mtDNA *in vivo*. However, this changes markedly in mice lacking Mpv17, where rGMP misincorporation increases, and in some cases becomes the preponderant embedded ribonucleotide. The change is associated with both mtDNA depletion and multiple deletions, suggesting that aberrant rGTP incorporation can reach levels that impair mtDNA replication, resulting in pathology.

## MATERIALS AND METHODS

### Animals and genotyping

CFW-Mpv17/J strain from Jackson laboratory (original stock number 002208) was backcrossed to C57Bl/6J background using the Marker-Assisted Accelerated Backcrossing (MAX-BAX^®^) provided by Charles River to generate 100% of C57Bl/6J within 10 generations. The congenic strain generated is identified as B6.CFW-Mpv17/J. Animals were genotyped by PCR, as described in (5). This research has been regulated under the Animals (Scientific Procedures) Act 1986 Amendment Regulations 2012 following ethical review by the University of Cambridge Animal Welfare and Ethical Review Body (AWERB) and the Francis Crick Institute, Mill Hill Laboratory (FCI‐MH).

### Mitochondrial isolation, nucleotide pool determination and DNA purification

Mitochondria were isolated from liver or brain and nucleotides were extracted from 500 μg aliquots of the mitochondrial preparation as previously described (5). Briefly, proteins from each mitochondrial pellet were precipitated with 0.5 M trichloroacetic acid and centrifuged at 20 000 × g for 5 min at 4°C. Supernatants were neutralized with 1.5 volumes of 0.5 M trioctylamine in Freon (1,1,2-trichlorotrifluoroethane) and centrifuged for 10 min at 10 000 × g at 4°C. The upper phase, containing nucleotides, was vacuum dried and stored at −80°C until analysis; dNTP concentrations were determined by a DNA polymerase-based method, as described in (16). For NTPs determination, nucleotides were extracted using methanol. Briefly, aliquots of isolated mitochondria were resuspended in cold 80% methanol, vortexed and incubated at −80°C for 4 h. Extracts were then centrifuged (16 000 × g for 10 min at 4°C) and supernatants were vacuum dried and stored at −80°C until analysis. NTP pools were estimated using T7 RNA polymerase incorporation of ^32^P-UTP (Perkin-Elmer), according to the manufacturer's instructions (Ambion) with PCR product corresponding to nucleotide position (np) 15890-168(T7) of murine mtDNA as template. One reaction (the reference) contained the other 3 NTPs, whereas other reactions lacked one of CTP, GTP or ATP but included the same lyophilate used in the DNA polymerase assay. For mtDNA purification isolated mitochondria were resuspended in 1.6 ml/g lysis buffer (20 mM HEPES pH 7.8, 75 mM NaCl, 50 mM EDTA and 200 μg/ml Proteinase K) on ice. After 10 min SDS was added to 0.25% (w/v) and incubated at room temperature for 50 min; mtDNA was isolated by phenol–chloroform extraction. For the detection of multiple deletions by Southern blot and mtDNA quantification, total DNA was extracted from 100 mg of brain, liver or heart. Minced tissues were lysed in 5 mM EDTA, 200 mM NaCl, 100 mM Tris, 0.2% SDS. After addition of 200 μg/ml of Proteinase K, samples were incubated at 56°C for 2 h and total DNA was extracted with phenol–chloroform.

### DNA modification, fractionation and Southern hybridization

For alkaline treatment 1 μg of mtDNA purified by sucrose gradient was treated either with 100 mM NaOH for the indicated times or RNase H2 (in-house preparation) and Mung Bean Nuclease (New England Biolabs). DNA samples were loaded directly on 1 X Tris-borate EDTA, 1% agarose gels or first denatured at 95°C for 3 min. For the detection of multiple deletions 3 μg of total DNA was digested with an mtDNA single cutter (BglII) and loaded on a 0.6% TBE–agarose gel. After electrophoresis, 250 mA for 4 h, gels were washed either in 10 × SSC (for denatured samples) or 400 mM NaOH for 15 min twice (for non-denatured samples), blot transferred to nylon membrane overnight and then UV cross-linked, total energy 1200 × 100 μJ/cm^2^. Membranes were pre-hybridized with 10 ml of hybridization solution (2 × SSPE, 2% SDS, 6 × Denhardts reagent, 5% Dextran Sulfate) for 30 min at 55°C. Riboprobes were synthesized from PCR products incubated with ATP, CTP, GTP and α-^32^P-UTP and T7 RNA polymerase at 20°C for 2 h (Ambion Maxiscript T7 In vitro Transcription Kit). After overnight hybridization at 55°C the membrane was washed repeatedly with 0.1 × SSPE, 0.5% SDS until there was no signal in the wash solution and exposed to X-ray film.

### Primer sequences used to generate PCR products for riboprobe synthesis

The T7 promoter sequence is underlined.

**Table:** 

nt (strand)	Sequence 5′-3′
300 (L)	ATTTCGTGCCAGCCACCGCGGTCATACGAT
T7 1000 (H)	TAATACGACTCACTATAGGGGTATGCTT
T7 14881 (L)	TAATACGACTCACTATAGGTCCCAGACATACTAGGAGACCCA
15800 (H)	AAGAACCAGATGTCTGATAAAGTTTC
15490 (H)	CTTGGGGAAAATAGTTTAATGTACG
T7 15750 (L)	TAATACGACTCACTATAGGATTAAACTTGGGGGTAGCTAAAC
16299 (H)	TTGTTAATGTTTATTGCGTAATAGAGT
340 (H)	GTTTGGGTTAATCGTATGACCG

### Quantification of mtDNA copy number

Relative amount of mtDNA was measured by real-time quantitative PCR as described in (5), with some modifications. Reactions were performed in triplicates on 384-well reaction plates (Applied Biosystems). Each PCR reaction had a final volume of 10 μl and contained 20 ng DNA, 5 μl of Power SYBR-Green PCR Master Mix (Applied Biosystems) and 0.5 μM of forward and reverse primers. Regions of Cox2 and App1 were amplified as mitochondrial and nuclear gene references, respectively, using the following primers: Mm-COXII-F: GAGCAGTCCCCTCCCTAGGA, Mm-COXII-R: GGTTTGATGTTACTGTTGCTTGATTT, Mm-APP1-F: CGGAAACGACGCTCT CATG, Mm-APP1-R: CCAGGCTGAATTCCCCAT. Changes in mtDNA amount were calculated using the 2^−ΔΔCt^ method (17) and represented as fold changes relative to the indicated control.

### Liver protein preparation and immunoblotting

Liver samples were homogenised in buffer consisting of 10 mM Tris–HCl pH 7.5, 1 mM EGTA, 75 mM sucrose, 225 mM sorbitol and 0.1% BSA, before lysis at 4°C in 50 mM Tris–HCl pH 7.4, 1 mM EDTA, 150 mM NaCl, 1% NP40, 0.1% SDS and 1 × protease inhibitor cocktail (Roche). Protein concentration was determined by Lowry assay (DC Reagent, Bio-Rad), and 5 μg of protein resolved on PAGE gels (Novex) before transfer to Imobilon-P membrane (Millipore). Following blocking with 5% non-fat dry milk in PBS with 0.1% (v/v) Tween-20, membranes were incubated overnight with the following primary antibodies: mouse anti-NDUFB8 (1:10 000, Abcam AB110242), mouse anti-COX IV (1:10 000, Abcam AB14744), rabbit anti-MPV17 (1:1000, Proteintech 10310-1-AP) or rabbit anti-TOM20 (1:20 000, Santa Cruz Biotechnology SC-11415). Membranes were incubated with HRP-conjugated anti-mouse or anti-rabbit secondary antibody (1:4000, Promega) in 5% non-fat dry milk in PBS with 0.1% (v/v) Tween-20, and visualised using enhanced chemiluminescence (ECL, G.E. Healthcare).

### EmRiboSeq library preparation

20 μg of total nucleic acid was treated with 2.5 μg RNase A (Invitrogen 46-7604) in 400 mM NaCl for 1 h at room temperature and fragmented using Covaris sonication (duty cycle: 10%, intensity: 5, cycles/burst: 200, time: 120 s at 4°C), after which the DNA was ethanol precipitated and used for library preparation, according to Ding *et al.* (15), with the following modifications: AMPure XP beads were removed after each clean-up step, to increase yield, and the final E-gel based size-selection step was omitted.

### Genomic mapping strategy

Mapping of reads was performed largely as previously described (15). To allow unique mapping of mitochondrial reads, high identity nuclear inserted mitochondrial derived sequences were N-masked in the mm9 reference genome. The reference chrM sequences was aligned by blast to the reference genome requiring high identity matches (match-score > 100, identity > 95%). All identified matching segments (excepting chrM itself) were N-masked in the reference. The same filtering approach was applied to reference genome segments matching the rDNA reference fragment and the rDNA reference sequence included with the mm9 genome for mapping. To allow unbiased mapping of reads over the ends of the artificially linearized chrM sequence in the reference assembly, a second version of the reference assembly was prepared containing the concatenated circularisation junction with 200nt flank on both sides and the corresponding sequences masked from the chrM sequence (the mm9chrMcirc genome). Ion Proton single end reads were clipped of residual adaptor sequence using cutadapt (options: overlap = 12 minimum-length = 30 and adaptor ‘a’ set to ATCACCGACTGCCCATAGAGAGG). After clipping, reads were aligned separately to the mm9 and mm9chrMcirc genomes using Bowtie2 (version 2.2.6). In the case of reads discordantly mapped between the mm9 and mm9chrMcirc genome the alignment with the highest mapping quality score were retained and coordinates of read 5′ ends transformed into mm9 coordinates. Except where otherwise stated, only reads with a phred-scaled map quality score of ≥30 were used for subsequent analysis. Read alignment extraction and filtering performed by Samtools (version 1.2). Embeded ribonucleotide positions were inferred as the nucleotide upstream and on the opposite strand of the read 5′ mapped end. Coordinate transforms and signal counting were performed using bedtools (version 2.26). The complete processing pipeline from FASTQ to Bed files of embedded nucleotides and derived figures is available at https://github.com/taylorLab/mito.

### Genomic composition

To calculate a relative rate of ribonucleotide incorporation we need to correct of genomic sequence composition. However, due to the repetitive nature of the genome and the necessity to filter on alignment quality, a substantial and compositionally biased portion of the genome cannot be mapped uniquely. Accounting for this, all segments of the reference genome that are uniquely mappable (GRCmappability100 mm9 track from UCSC) were extracted (bedtools getfasta) and their nucleotide and trinucleotide compositions calculated. Trinucleotide context was calculated for each identified riboincorporation site, where the ribo was the central of the three nucleotides on the ribo-containing strand.

### Sequencing library preparation and analysis

For the sequencing analysis, mouse brain mtDNA was purified from sucrose-gradient isolated mitochondria as above. Purified mtDNA was fragmented prior to library preparation as previously (5). 200 bp paired-end DNA libraries were prepared using the Illumina TruSeq LT kit and run on the MiSeq. Sequencing data (FASTQ) files were mapped to the MT mm9 assembly and the reads mapped using BWA (Burrows-Wheeler Aligner) software (Li (2013)) Aligning sequence reads, clone sequences and assembly contigs with BWA-MEM. *arXiv:**1303.3997*), version bwa-0.7.8, bwa-mem algorithm with default parameters. The mapped read (sam) files were then converted to bam format using samtools version 0.1.19 (18). The reads in each bam file were sorted and indexed using samtools. Finally, the two bam files from different sequencing runs for each sample were merged using Picard tool MergeSamFiles. Coverage and mutation loads were calculated as previously (5).

### Statistical analysis

Data analysis and figure plotting was performed in R (version 3.4.1) utilising standard libraries and the rollapply function from the Zoo package (19). A complete set of R analysis and plotting code is available at https://github.com/taylorLab/mito. For mtDNA and dNTPs pool size, data are expressed as the mean ± standard error of the mean (SEM). Group means were compared using parametric t-test or non-parametric Mann-Whitney test. *P* < 0.05 was considered as statistically significant.

## RESULTS

### Ribonucleotide incorporation in mtDNA is substantially higher in solid tissues than cultured cells

**Figure 1.**
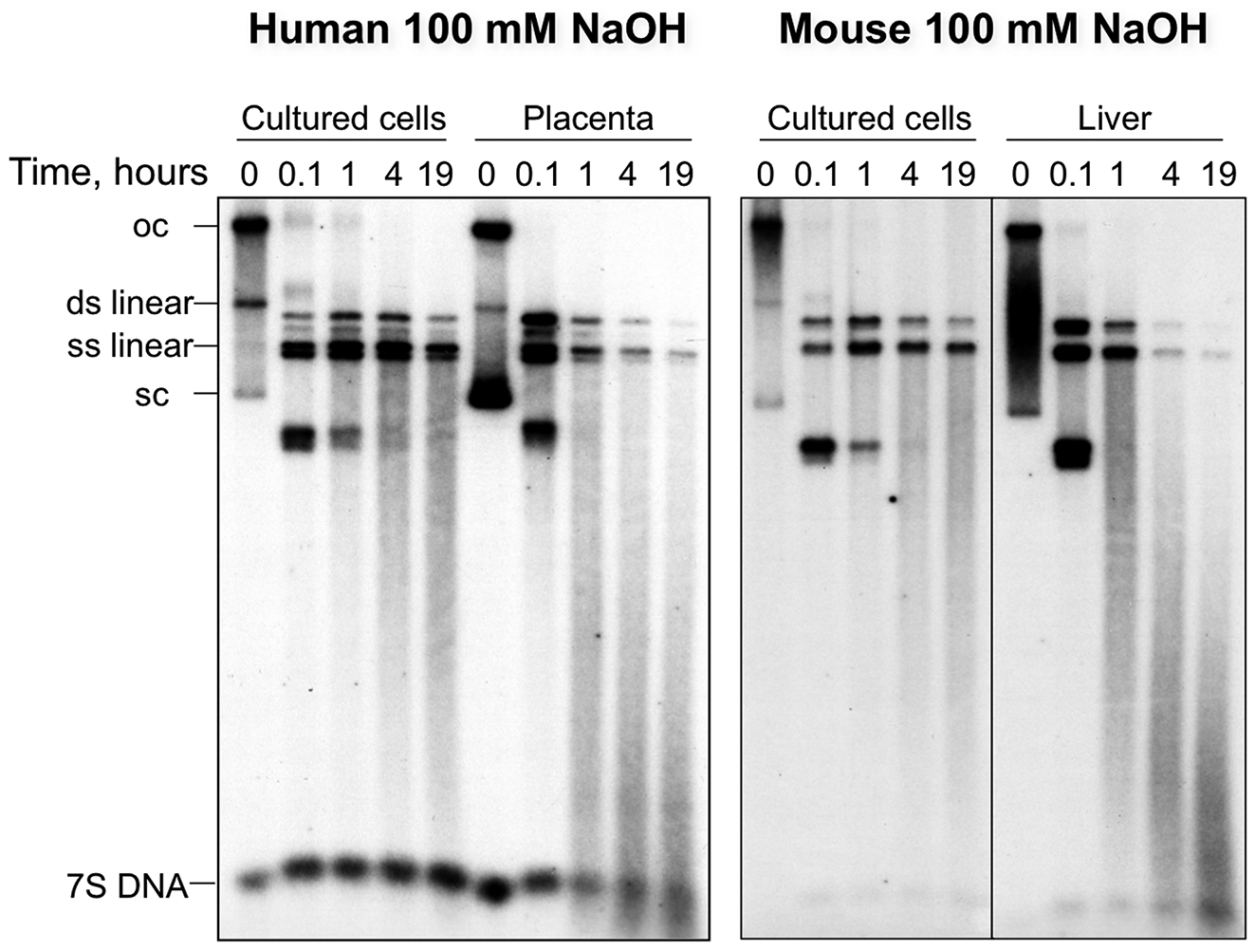
Mammalian mtDNA from solid tissues is more sensitive to alkali than that of proliferating cultured cells. DNA from isolated mitochondria of human 143B cells, human placenta, murine LA9 cells and mouse liver was fractionated by 1D-AGE, after exposure to 100 mM sodium hydroxide (NaOH) for the indicated times, and blot hybridized to radiolabeled probes corresponding to segments of human (16 343–151) or murine (15 007–15 805) mtDNA. oc – open circlular, sc – supercolied, and ds – double stranded, ss – single stranded mtDNA. Treatments with type 2 RNase H fragmented the murine liver mtDNA to a similar extent (Supplementary Figure S1), indicating that most alkali sensitive sites are rNMPs and not abasic sites.

Most studies of the presence of ribonucleotides in mtDNA have used material from cultured cells (7,9,12). To determine whether the ribosubstitution of mammalian mtDNA is a general phenomenon and therefore also present *in vivo*, human placenta and murine solid tissues, as well as cultured cells (mouse LA9 and human 143B osteosarcoma cells) were subjected to alkali or type 2 RNase treatment. The assays confirmed the presence of numerous rNMPs scattered throughout the molecules (Figure 1, Supplementary Figure S1), and showed that the fragmentation was more marked for the mtDNA of solid tissues than for proliferating cells (Figure 1), indicating that ribosubstitution of mtDNA is considerably more prevalent *in vivo*, in post-mitotic tissues.

### rAMP dominates as the most abundant ribosubstitution throughout both strands of mtDNA of murine solid tissues

**Figure 2.**
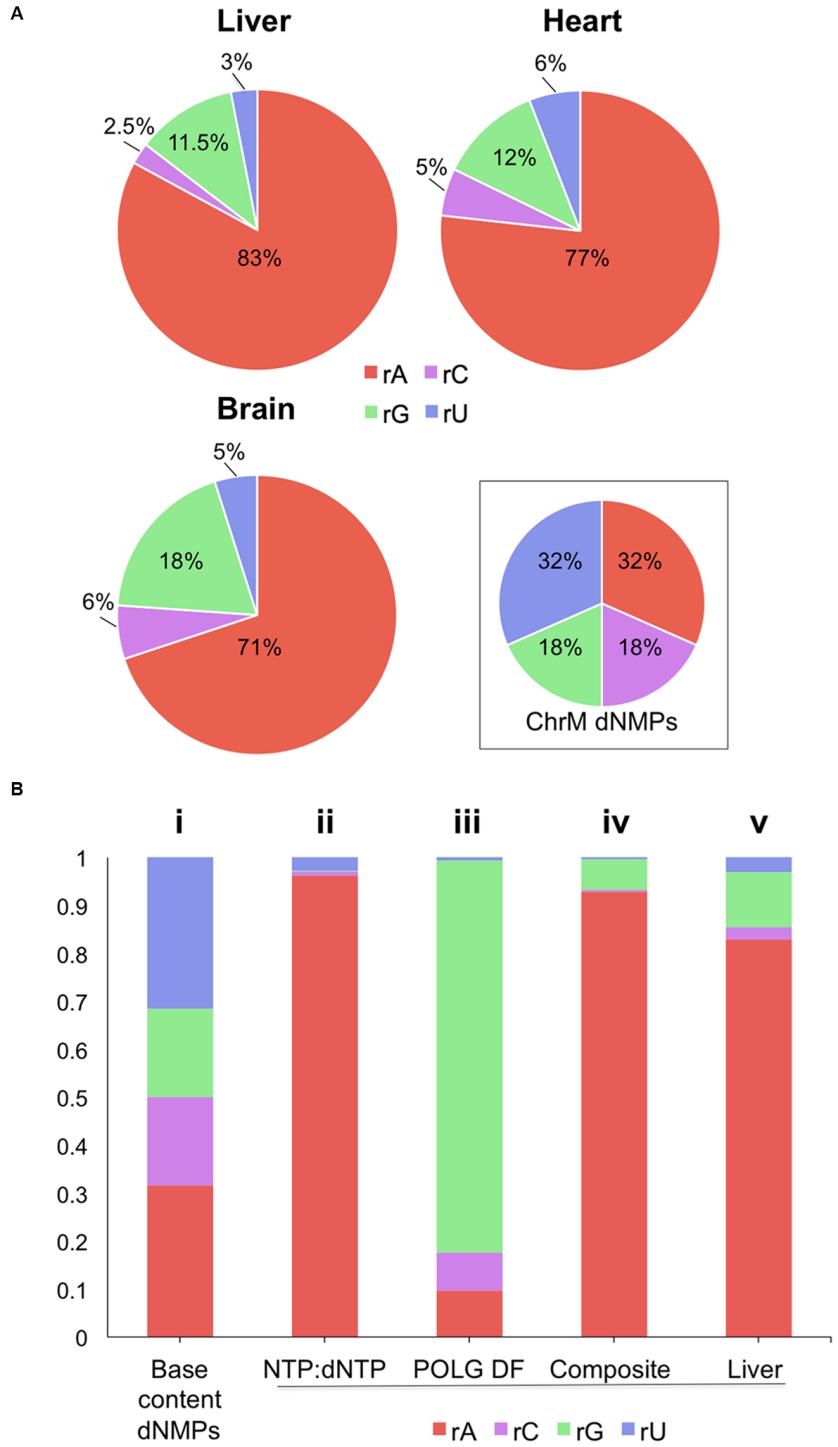
rAMP is the dominant ribonucleotide in mtDNA of murine solid tissues, the combined result of nucleotide concentrations and the properties of POLG. (**A**) The proportion of each of the embedded rNMPs in murine liver, brain and heart mtDNA and the base composition of the ‘primary sequence’ of the mouse mitochondrial genome (ChrM). (**B**) Expected proportions of embedded rNMPs if the sole determinant were: (i) the mtDNA base composition, (ii) the rNTP/dNTP ratio in liver mitochondria (derived from (20)),  (iii) the discrimination factor (DF) of the mitochondrial DNA polymerase (POLG) (derived from (21)), both adjusted for the mtDNA sequence; or (iv) if rNMP incorporation was a function of both the POLG DF and the rNTP:dNTP ratio. This ‘composite’ is most similar to the observed proportions for liver (v).

To determine the identity and distribution of ribonucleotides in mtDNA we performed deep sequencing of emRiboSeq libraries of purified murine liver mtDNA, prepared according to (15) (see Supplementary Figure S2 for details and validation). Alignment of tens of millions of reads to the mitochondrial genome revealed marked differences among the four ribonucleotides. Overall more than 80% of all ribonucleotides embedded in murine liver mtDNA identified were rAMPs, disproportionate to the number of adenine bases present in the murine mitochondrial genome; and rAMPs also outnumbered the other rNMPs in brain and heart mtDNA (Figure 2A). Thus, rAMPs are the predominant ribosubstitution event in the mtDNA of solid tissues. In contrast, analysis of nuclear DNA reads present in the same emRiboSeq libraries showed that there was no appreciable bias towards a particular rNMP in the nuclear genome of murine liver or that of mouse embryonic fibroblasts (MEF) (Supplementary Figures S3A and B). MEF mtDNA also did not display the pronounced preponderance of rAMPs seen in solid tissue (Supplementary Figure S3C). In mtDNA, the rNMPs were widely distributed across both strands (Supplementary Figure S4A), with the precise distribution being influenced partially by the trinucleotide context (Supplementary Figure S4B and S4C). Although there appeared to be some pronounced regional biases, these were largely attributable to low mapping quality, owing to truncated reads (Supplementary Figure S5).

**Figure 3.**
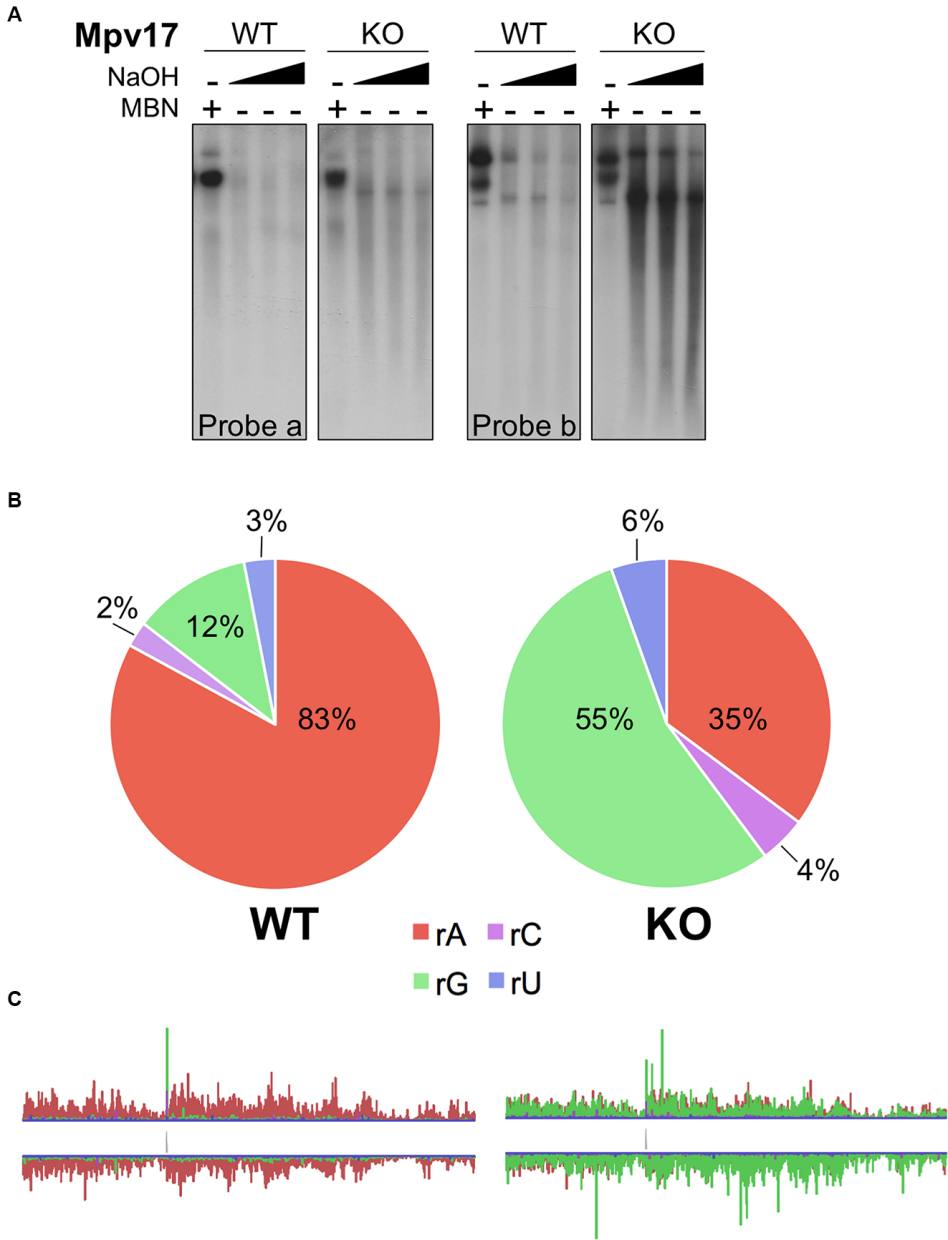
rGMP replaces rAMP as the predominant embedded ribonucleotide in mtDNA in the liver of the Mpv17 knockout mouse. (**A**) Alkali (NaOH) fragmentation of liver mtDNA from wild-type (WT) and Mpv17 knockout (KO) mice analyzed by agarose gel electrophoresis and Southern hybridization with probes to np 4000–8120 (probe a) and 14881–16299 (probe b); MBN – mung bean nuclease treatment. (**B**) The proportion and (**C**) the distribution (enlarged in Supplementary Figures S4 and S8) of the rNMPs in murine liver mtDNA with (WT) or lacking (KO) the *Mpv17* gene.

The heavy bias towards rAMPs in solid tissue is readily explained by the high ATP level in mitochondria (20), predicting a skew away from equality and heavily towards rAMP incorporation (Figure 2B–i compared to 2B-ii). Besides the nucleotide pool sizes, the incidence of embedded ribonucleotides in mtDNA is affected by the ability of the replicative polymerase to discriminate deoxyribonucleotides from ribonucleotides, the discrimination factor of the enzyme (21). In the case of mtDNA polymerase POLG, this measure would predict rGMP to be the most frequently incorporated rNMP (Figure 2B-iii). The composite of the two predictions yields a pattern very much in line with the results of the emRiboSeq analysis (Figure 2B-iv versus B-v) and thus the representation of the different ribonucleotides in mtDNA of murine solid tissues (Figure 2A) can largely be explained by the concentrations of the different rNTPs relative to dNTPs inside mitochondria, coupled with the ability of POLG (and the other DNA polymerases in mitochondria (22,23)) to discriminate each dNTP from its rNTP counterpart.

### rGMP replaces rAMP as the predominant ribonucleotide embedded in mtDNA in Mpv17 deficient mouse liver

Having established that the rNTP:dNTP ratio is an important determinant of the ribosubstitution rate of mtDNA, we predicted that any increase in this ratio will lead to an increase in the ribonucleotides incorporated in mtDNA. This hypothesis was tested in the liver of the Mpv17 deficient mouse, which displays OXPHOS deficiency, decreased mitochondrial dNTP pools and mtDNA depletion ((5) and Supplementary Figure S6). The total amount of embedded rNMPs was similar to that of controls in the livers of Mpv17 knockout mice, based on the alkali fragmentation assay (Figure 3A), but there was a substantial increase in rGMP incorporation based on emRiboSeq analysis (Figure 3B). These observations could be explained by an increased rGTP:dGTP ratio, as liver mitochondria of the Mpv17 null mouse show a marked reduction in the level of dGTP, alongside an expected decreased ATP:dATP ratio owing to reduced mitochondrial ATP production as a result of OXPHOS deficiency (Supplementary Figure S6). Notwithstanding this, findings from other tissues of the Mpv17^−/−^ mouse indicated that this model needed to be further refined.

### Normal mtDNA copy number and dGTP levels, but elevated rGMP incorporation in Mpv17 deficient brain

**Figure 4.**
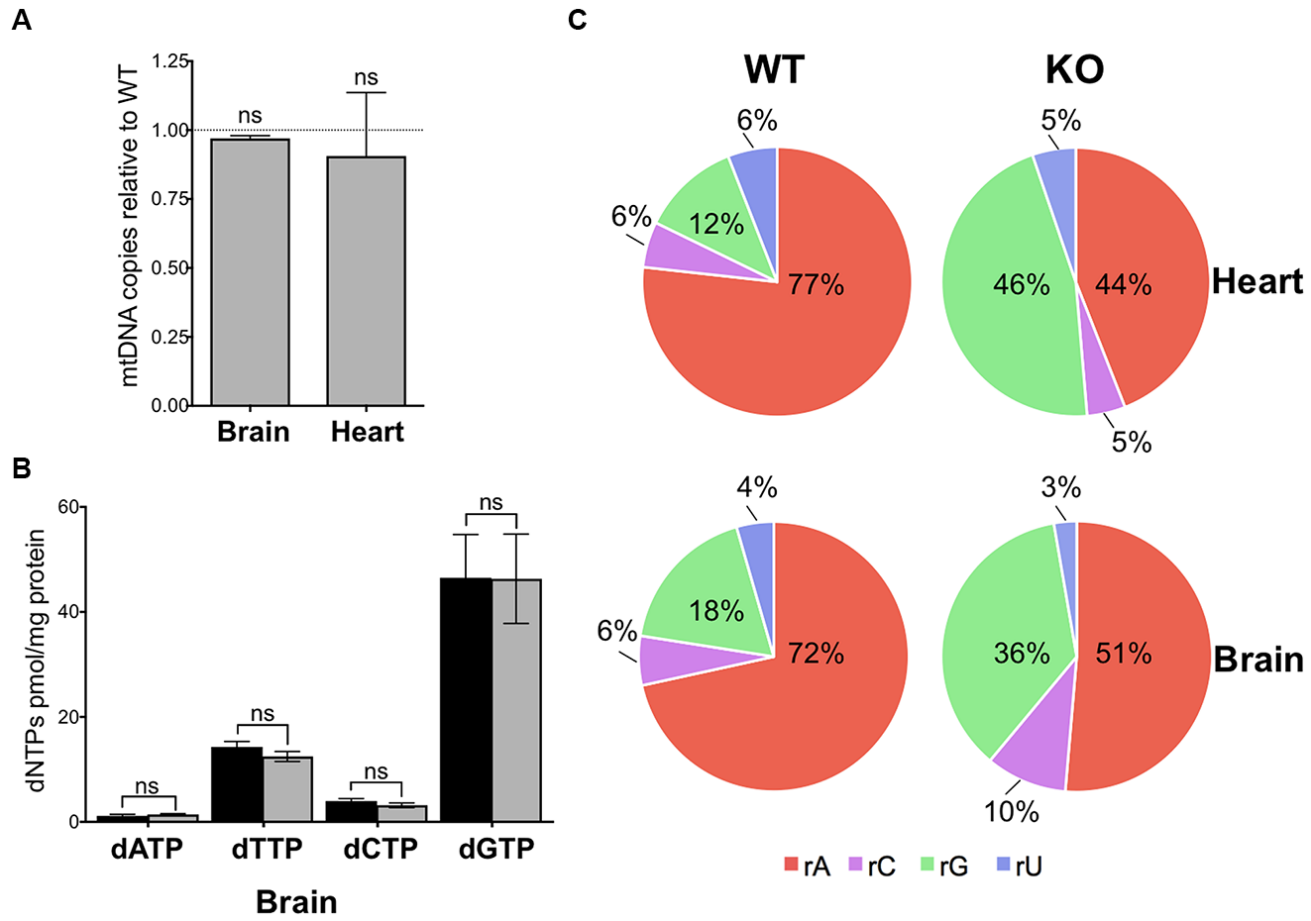
Elevated embedded rGMPs in mtDNA of brain and heart from Mpv17 null mice, without altered dNTP pools or mtDNA copy number. (**A**) Mitochondrial (mt) DNA copy number in brain and heart of Mpv17^−/−^ mice (gray) relative to controls (WT), n = 3 independent experiments, t-test; and (**B**) Brain mitochondrial dNTP pools in control (black) and Mpv17^−/−^ (gray) animals of 8–10 weeks of age. P values were obtained using Mann-Whitney test (n = 4). Not significant (ns) P > 0.05. Data are expressed as mean ± SEM. (**C**) Relative frequency of incorporated ribonucleotides of heart and brain mtDNA of Mpv17 KO mice of 2–3 months of age determined by emRiboSeq represented as pie charts; rA – rAMP, rC – rCMP, rG – rGMP, rU – rUMP/rTMP.

Brain and heart of the Mpv17 ablated mice maintained mtDNA copy number at normal levels (Figure 4A), and dNTP concentrations in the brain mitochondria were comparable to controls (Figure 4B). Furthermore, sequencing of brain mtDNA indicated no increase in the error rate for dNMPs (Supplementary Table S1). However, the proportion of rGMPs embedded in the mtDNA increased 2–4-fold in brain and heart of the Mpv17^−/−^ mice (Figure 4C). Although raised rGTP levels could also lead to an elevated rGTP/dGTP ratio and consequent increase in rGMP incorporation, there was no significant increase in the concentration of rGTP in mitochondria of either an affected (liver) or an unaffected tissue (kidney), based on an RNA polymerase extension assay (Supplementary Figure S7). These findings suggest that high levels of embedded rGMPs in mtDNA occur as a consequence of Mpv17 deficiency in every tissue, irrespective of the overall nucleotide pool sizes.

### Late onset multiple deletions of mtDNA in brain of Mpv17 null mice

**Figure 5.**
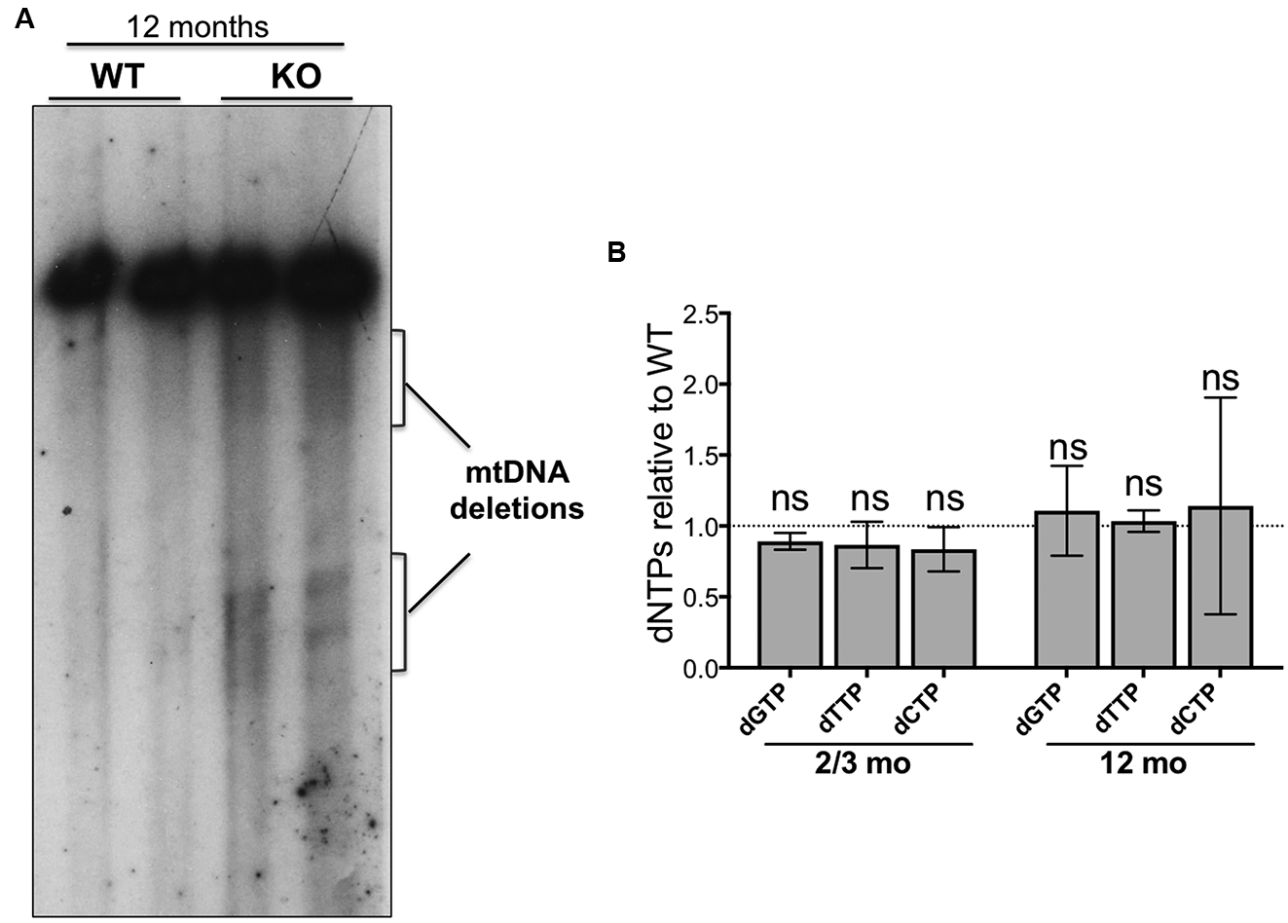
Multiple deletions that are synonymous with DNA replication stalling are detected in the brain of Mpv17 null mice of 12 months of age. (**A**) Southern blot of brain DNA of mice aged 1 year hybridized with a probe to np 14881–16299 of the murine mitochondrial genome. Wild-type mice (WT) and Mpv17 knockout animals (KO). Note the more prominent smear of linear deleted molecules below the full length mtDNAs in the KO samples. (**B**) dNTP levels in brain mitochondria of Mpv17 KO mice aged 2–3 months or 10–12 months, expressed relative to age matched controls, *n* = 4. *P* values were obtained using Mann-Whitney test (NS, not significant *P*>0.05).

Elevated rGMP incorporation is a pronounced mtDNA abnormality of Mpv17 deficient mice (Figures 3 and 4); if it is pathological, mtDNA abnormalities less extreme than depletion may occur in tissues other than liver. Based on the human disorders, brain is expected to be the second most vulnerable tissue of the Mpv17 ablated mouse, and late-onset multiple deletions of mtDNA the likely defect (6,24,25). Therefore, mtDNA of 12-month-old Mpv17^−/−^ mice was analyzed by Southern hybridization and multiple deletions of mtDNA, not evident in age-matched littermates controls, were detected (Figure 5A), while dNTP levels in brains of both young and old Mpv17^−/−^ animals remained within the normal range (Figure 5B). We therefore establish that in spite of normal overall dNTP levels, rGMP incorporation in brain mtDNA is elevated in Mpv17 knockout mice, and that this is accompanied by late-onset mtDNA deletions.

## DISCUSSION

Our analysis of embedded ribonucleotides in mtDNA of solid tissues indicates that although the discrimination factor of the mitochondrial DNA polymerase contributes to the observed ribonucleotide incorporation patterns (12), the rNTP:dNTP ratio is the single most important determinant in the ribosubstitution of mature mammalian mtDNA (Figures 1–3). Moreover, the markedly lower ribosubstitution frequency of mtDNA of cultured cells compared to solid tissues (Figure 1) can also be attributed to the rNTP:dNTP ratio, as cycling cells require high concentrations of dNTPs to support nuclear DNA replication (26). Evidently, the presence of riboadenosines in the mtDNA of solid tissues is well tolerated. It is plausible that over the course of time, evolution may have exploited the presence of riboadenosines in mtDNA of solid tissues. For example, as embedded rAMPs produce subtle changes in the structure of the phosphodiester backbone (27) their presence might facilitate or discourage interactions with nucleic acid binding proteins.

### MPV17 function, rGMPs and disease mechanism

The normal level of dGTP in the brain of the Mpv17 knockout mouse argues against the protein being involved in dNTP synthesis or import into mitochondria. Nevertheless, the weight of evidence still points to guanosine metabolism being critical to the MPV17-related disorder. Clinically, MPV17 deficiency is most similar to diseases caused by mutations in deoxyguanosine kinase (DGOUK), with both giving rise to hepato-cerebral syndromes (6,28). Moreover, zebrafish lose their stripes of crystallized guanine when Mpv17 is scarce (29). The data presented here further contribute that rGMP incorporation is a general aberrant feature of mammalian mtDNA in the absence of Mpv17 (Figures 3 and 4) and strongly supports the earlier conclusion that alteration of nucleotide homeostasis underlies the pathogenesis of MPV17-related disease (5). Three mechanisms of action can be envisaged for Mpv17 to explain the high level of rGMP in mature mtDNA, all of which involve the protein facilitating the supply of (pristine) dGTP to the replisome: i) Mpv17 alters the discrimination properties of POLG, leading to increased misincorporation of rGTP, although there is currently no evidence that the two proteins interact (30); ii) sanitation of dNTPs is an important function for nuclear DNA replication (31), hence, Mpv17 might sanitize dGTP, and in its absence, while dGTP levels are normal, many more damaged forms are presented to the replicative DNA polymerase, increasing the use of rGTP as an undamaged alternative; or iii) Mpv17 influences the concentration of dNTPs in the vicinity of the replisome, in particular dGTP. One or more proteins are expected to have such a function, because a means of concentrating dNTPs where they are needed would greatly reduce the demand on deoxynucleotide biosynthesis for mtDNA replication and minimize misincorporation of ribonucleotides, while simultaneously reducing dNTP interference with the many processes utilizing rNTPs.

**Figure 6.**
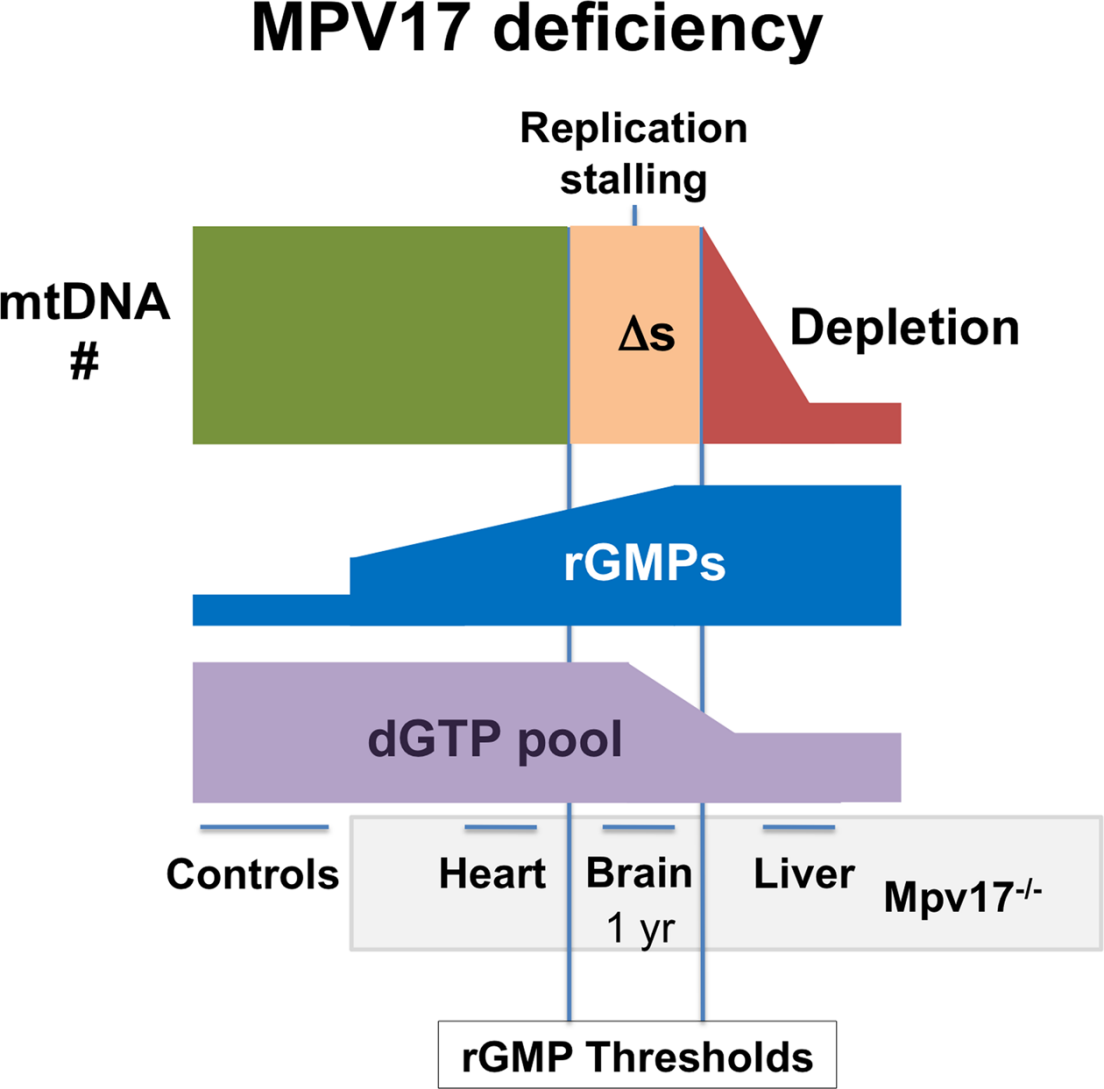
Model of disease mechanism for MPV17 deficiency. Loss of function of MPV17 in humans and mice results in multiple deletions and depletion of mtDNA. In mice, high levels of embedded rGMPs are a pronounced mtDNA abnormality that appears to be an unavoidable consequence of Mpv17 deficiency, which, we propose, is the result of changes in the local ratio of rGTP/dGTP. The exact level of embedded rGMPs is expected to depend on tissue specific features of nucleotide metabolism and the rates of mtDNA synthesis and turnover, but whenever rGMPs exceed a certain threshold it impedes DNA replication causing stalling, manifesting as multiple deletions (Δs), as seen in the brain of 12-month old Mpv17^−/−^ mice. Worsening of the problem would be expected to lead eventually to complete replication arrest. Therefore, we interpret the lowering of the dGTP and dTTP pools (this report and (5)), as an adjustment of dNTP pools towards equality, to slow replication and improve fidelity (5,34), that allows some functional mtDNA molecules to be maintained indefinitely, as seen in the liver of Mpv17 knockout mice (35).

That elevated rGMP is the primary abnormality of the mtDNA of Mpv17 deficient mice suggests that these incorporated ribonucleotides are fundamental to the disease. Even a single rGMP in a template slows the rate of synthesis of POLG *in vitro* (21), and embedded rGMP distorts the double helix substantially and affects melting temperature (27,32). Each of these factors could increase the probability of DNA replication stalling, and thus at the high rGMP levels reached in some solid tissues or cell types lead to an accumulation of stalled replication intermediates, evident as multiple deletions of mtDNA. In extremis, dNTP pools may be adjusted to slow replication and mitigate the stalling, but at the cost of mtDNA depletion (illustrated schematically in Figure 6).

The striking changes in ribonucleotide incorporation in mtDNA associated with Mpv17 deficiency raise the question of whether this abnormality might explain other currently obscure causes of mtDNA disease. For example, mutations in the adenine nucleotide transporter (ANT1) were the first identified cause of mtDNA deletions (33), yet a molecular explanation is still lacking. If the pathological ANT mutants lead to elevated ATP levels, particularly in the vicinity of replicating mtDNAs, then this could push embedded rAMPs to deleterious levels. Hence, many aspects of mitochondrial DNA disorders need to be reconsidered in light of the fact that a striking abnormality, embedded ribonucleotides, has hitherto been largely overlooked.

## AVAILABILITY

GEO accession number: GSE103429.

ENA (Next generation sequencing):

Accession Unique Name

Sample ERS1906206 (SAMEA104281228): CM1

Sample ERS1906207 (SAMEA104281229): CM2

Sample ERS1906208 (SAMEA104281230): CM3

Sample ERS1906209 (SAMEA104281231): CM4

Sample ERS1906210 (SAMEA104281232): CM5

Sample ERS1906211 (SAMEA104281233): CM6

The complete processing pipeline from FASTQ to Bed files of embedded nucleotides and derived figures is available at https://github.com/taylorLab/mito

## Supplementary Material

Supplementary Data
