# Supplementary material for: Aberrant ribonucleotide incorporation and multiple deletions in mitochondrial DNA of the murine MPV17 disease model
Source: Nucleic Acids Res. 2017 Nov 2;45(22):12808–15. doi: 10.1093/nar/gkx1009 (PMC5728394; doi:10.1093/nar/gkx1009)
Supplement: Supplementary Data [file gkx1009_supp.pdf]

Aberrant ribonucleotide incorporation and multiple deletions in mitochondrial DNA of the murine MPV17 disease model

Supplementary Data

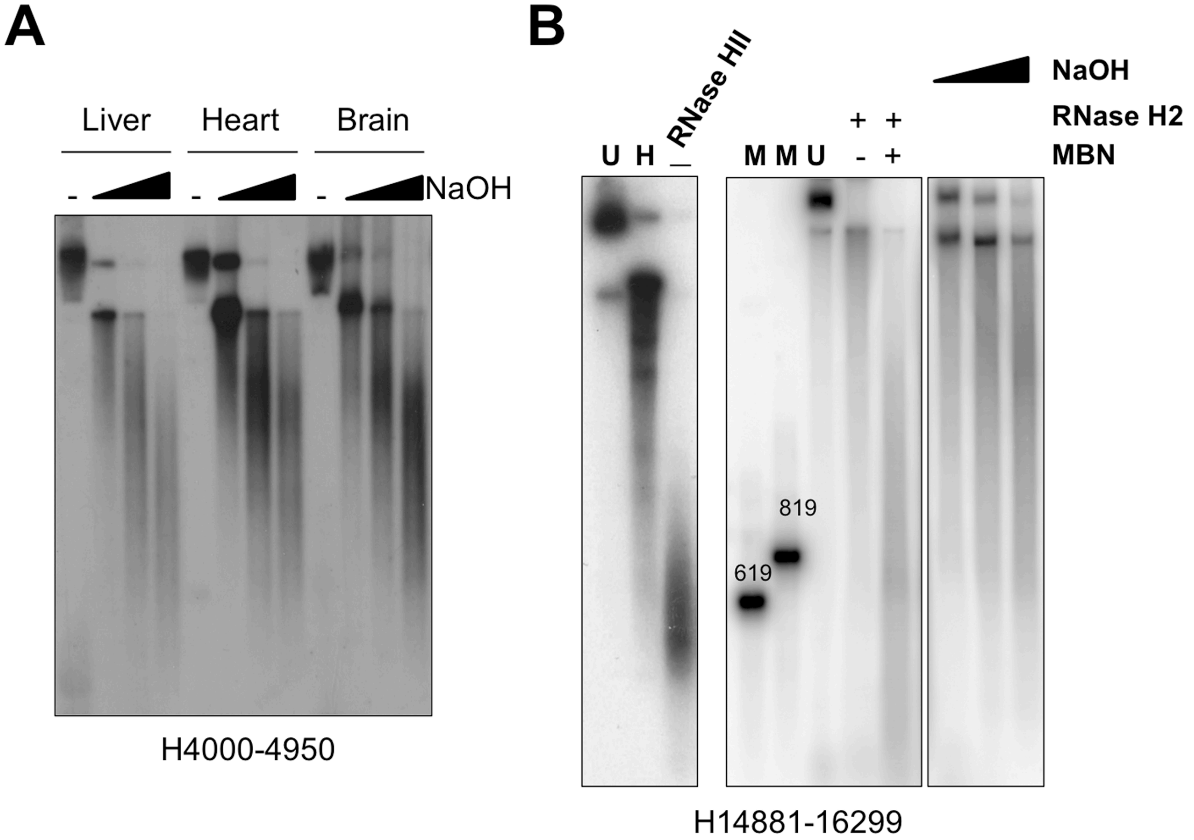

Figure S1. Murine mtDNA of solid tissues is fragmented by alkali or RNase H2. (A) DNA from isolated mitochondria of mouse liver, heart or brain was incubated with 0, 50, 100 or 200 mM NaOH at 37°C for 1 h and fractionated by 1D-AGE, prior to Southern hybridization to a H-strand specific riboprobe corresponding to np 4,000-4,950 of murine mtDNA. **(B)** Purified murine liver mtDNA was fractionated by 1D-AGE, after exposure to heat, RNase HII or a combination of RNase H2 and mung bean nuclease (MBN), which cleaves single-stranded DNA at nicks in duplex DNA. The DNA was blot hybridized to a riboprobe complementary to the region of H-strand murine mtDNA spanning np 14,881-16,299. Left gel image: U – untreated, H – heat, 95°C 3 min, RNase HII (10 U, New England Biolabs), 37°C, 30 min. Right gel image (in two parts with intervening lanes omitted): marker lanes with DNA fragments of 619 and 819 nucleotides followed by mtDNA 1) uncut; 2) 0.5 μM RNase H2, 37°C, 2 h; 3) 0.5 μM RNase H2, 2 h MBN (5U) 3 h; 4) 100 mM NaOH; 5) 150 mM NaOH; 6) 200 mM NaOH 30 min.

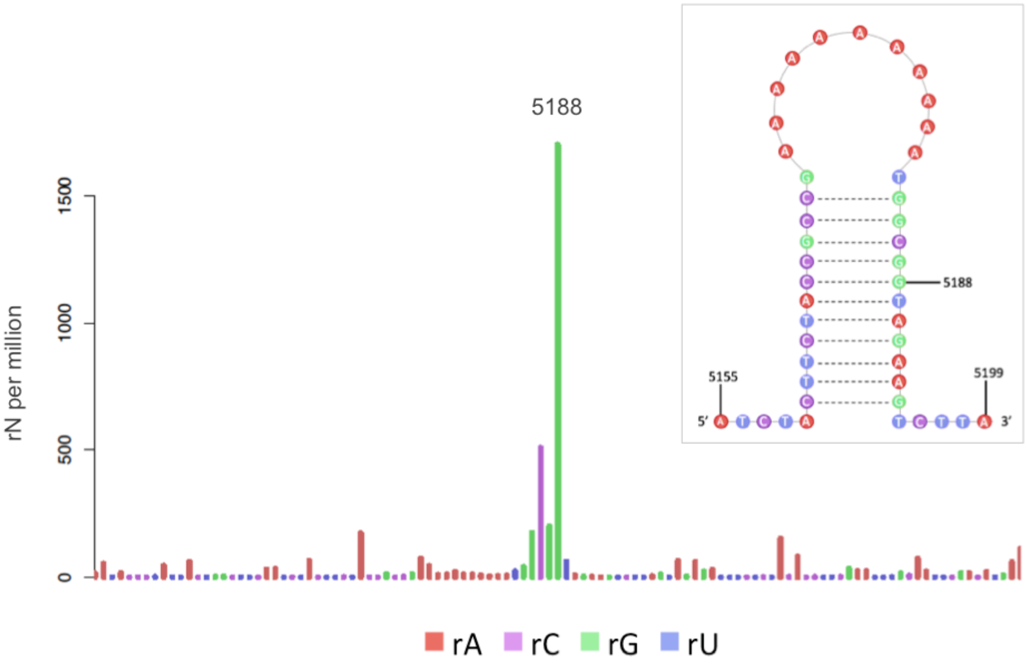

Figure S2. Distribution and frequency of embedded rNMPs for the region encompassing the prominent site of initiation of second strand DNA synthesis, Ori-L. To determine the distribution and relative abundance of the four ribonucleotides in mtDNA, EmRiboSeq (1) was applied to mtDNA from tissues and cells of the mouse. Previously, the prominent site of initiation of second strand DNA synthesis known as Ori-L, was shown to be an alkali sensitive locus mapping to np 5,188 of mouse mtDNA (2), and ribonucleotide retention in the vicinity of Ori-L has been demonstrated by other methods (3). Based on EmRiboSeq, the single most frequent ribosubstitution of murine mtDNA is a guanosine at np 5,188, which confirms the location of Ori-L, and validates the technique. The example shown is murine liver mtDNA. It is important to note that this does not necessarily mean that there is always a single rGMP at the site of RNase HII cleavage, as any closely spaced rNMPs on the same molecule will escape detection, because EmRiboSeq is unable to distinguish between one or more consecutive ribonucleotides. This is a limitation of all ribosubstitution detection methods (4), including Pu-seq (5), and is due to the nature of hydrolysis at rNMPs in DNA. That said, patches of four or more ribonucleotides are known to be rare as RNase HI cleaves isolated mammalian mtDNA at few, if any, sites in most molecules (6) (3). Inset: the position of the prominent rNMP at np 5188 mapped to the predicted stem-loop structure for Ori-L (7). Sequences in the nuclear DNA matching the mitochondrial genome (so-called NUMTs) were not expected to cause any significant interference because rNMPs are present at much lower density in nuclear than mitochondrial DNA (8), and consistent with this expectation the rNMP data for mtDNA were not evidently affected by the proportion of the total number of reads aligning to mitochondrial DNA (which varied from 4-98% mtDNA).

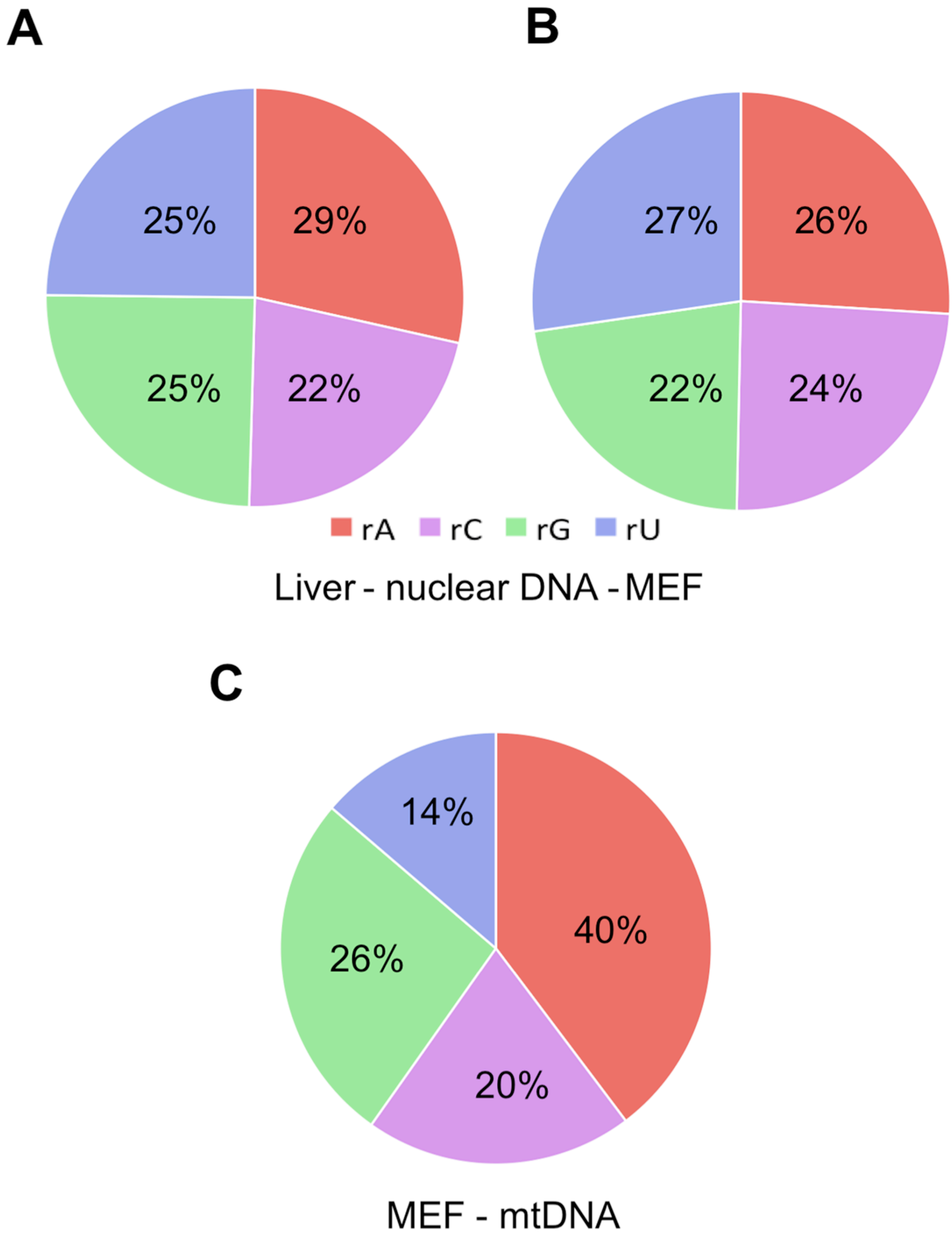

Figure S3. There is no skew toward rAMP in murine nuclear DNA, and mtDNA of murine cultured cells has proportionally fewer rAMPs than that of solid tissues. The proportions of the four rNMPs in the nuclear DNA of (**A**) murine liver (**B**) mouse embryonic fibroblasts (MEF); and in (**C**) the mtDNA of MEFs.

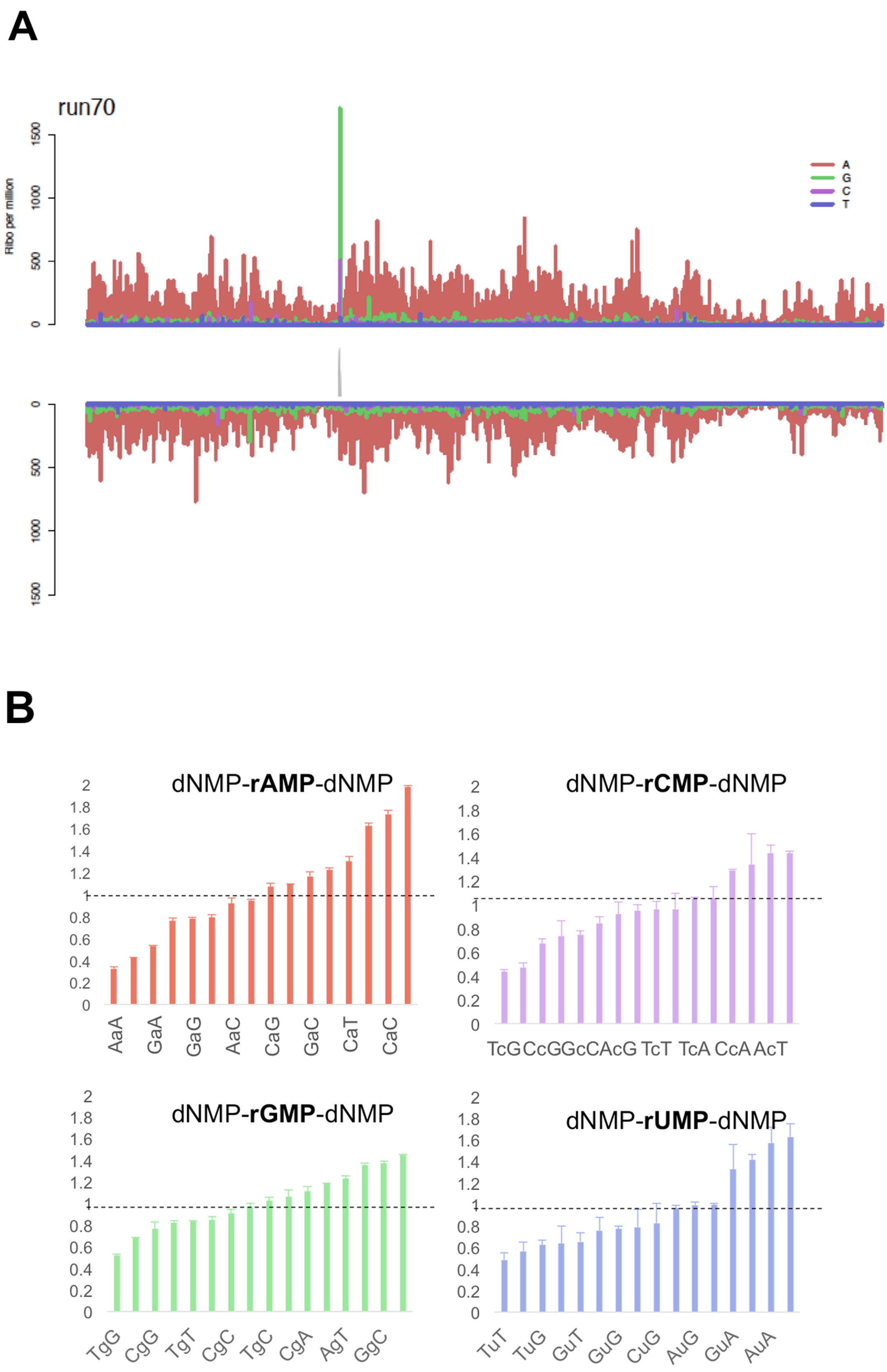
A
run70
Ribo per million
1500
1000
500
0
A
G
C
T
B
dNMP-rAMP-dNMP
AaA GaA GaG AaC CaG GaC CaT CaC
dNMP-rCMP-dNMP
TcG CcG GcC AcG TcT TcA CcA AcT
dNMP-rGMP-dNMP
TgG CgG TgT CgC TgC CgA AgT GgC
dNMP-rUMP-dNMP
TuT TuG GuT GuG CuG AuG GuA AuA
2
1.8
1.6
1.4
1.2
1
0.8
0.6
0.4
0.2
0

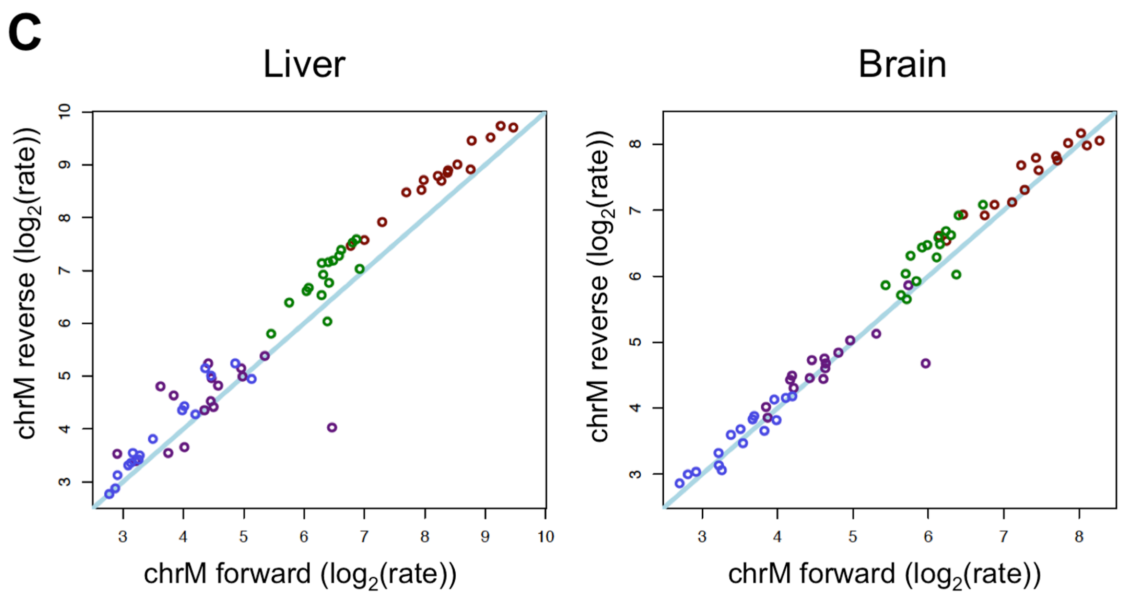

Figure S4. Trinucleotide context analysis indicates flanking deoxynucleotides influence rNMP misincorporation. (A) The distribution of the rNMPs in murine liver mtDNA. The circular mitochondrial genome is displayed linearly with the first nucleotide far left and np 16,299 to the far right. Upper plots are H-strand, lower plots L-strand. The total of the all histogram bars over both strands for the WT plot sum to 1 million. **(B)** A ribonucleotide may be more or less likely to be incorporated in DNA depending on the preceding dNMP or the following dNTP and this trinucleotide context was shown to be influential in determining the overall ribosubstitution rate in vitro (9). Trinucleotide context analysis of the EmRiboSeq datasets is represented as the frequency of each ribonucleotide (rNMP) with respect to all the possible flanking dNMPs (64 triplets) normalized for the occurrence of each triplet sequence in the murine mitochondrial genome and expressed relative to the mean (set to 1) of all 16 possible triplets for each rNMP. The range from least to most frequent triplet was almost five-fold for rAMP and rUMP, but less than three-fold for rGMP. Although rUMP was most likely to be followed by dAMP, this should be seen in the context of the very low numbers of rUMPs in murine liver mtDNA; whereas the triplets CaC, TaC and TaT that were 1.6-1.8 times more frequent than expected represent hundreds more sites of ribosubstitution because of the high levels of rAMPs in the liver mtDNA. Other points to note: ATP is more often incorporated after dCMP or dTMP residues; there is a general bias against incorporating the same base after a rNMP, e.g. AaA; and triplet symmetry is favored in a number of cases (CaC, TaT, AcA, AuA and GcG). Although repair systems may contribute to the observed biases, no mechanisms have been identified that allow targeted removal of embedded ribonucleotides from mtDNA; therefore, these features are most likely attributable to POLG and any ancillary factors of the mitochondrial replisome. (**C**) Ribonucleotide rates per trinucleotide are similar between the two chrM strands. Each site of the chrM genome was categorized as one of 64 trinucleotides and the frequency at which the central nucleotide was an rN was calculated for each trinucleotide category for the forward (light) as well as the reverse (heavy) strand. Each point represents one of the 64 trinucleotides, colored according to the identity of the central rN (A, U, C, G). The diagonal (light blue solid line) corresponds to equal rates between strands. Both strands show the same rate distribution. One prominent outlier (rC, purple spot) indicates discordance between

strands in the rate of incorporation in the GcG nucleotide context. This is attributable to the GcG signal being dominated by a single site at Ori-L (nucleotide position 5186 on the light strand; Fig S2) combined with the low rN incorporation rate in this context through the remainder of the mitochondrial genome. In contrast the ratio distorting effect of rG in a GgT context at position 5188, which shows even greater ribonucleotide incorporation than position 5186 (Fig S2), is less marked. This because, through the mitochondrial genome, GgT shows both a greater median rN signal and 4.7-fold (436 versus 93) more sites than the GcG context, diluting the effect of the single site at the Ori-L overall strand ratio.

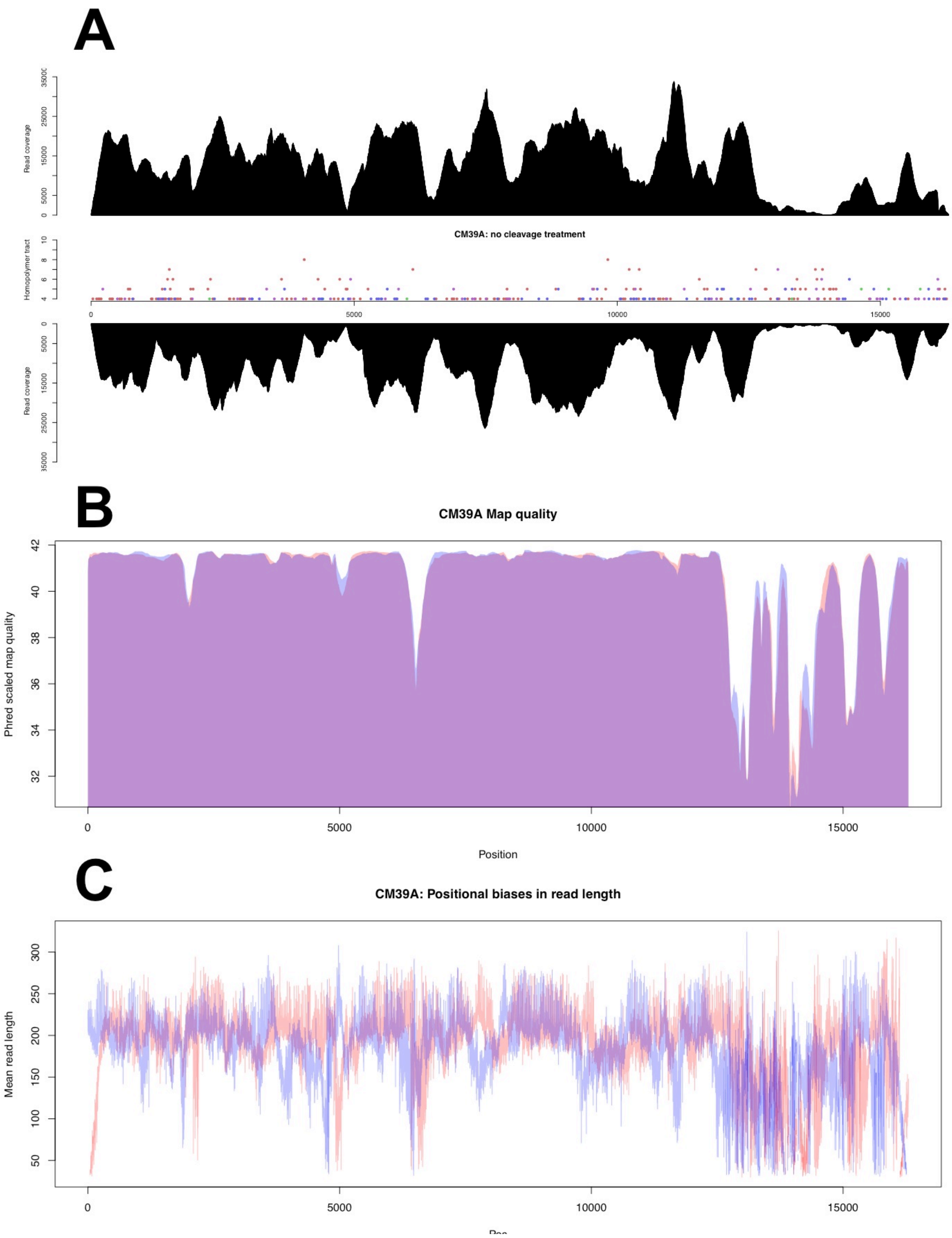

Figure S5. Low and high coverage regions are not indicative of low and high ribonucleotide incidence. (A) Pronounced peaks and troughs similar to those of the RNase H2 treatments were also seen when a library of mtDNA was prepared without the use of RNase H2. **(B)** The regions of lowest coverage (deepest troughs) correspond to low mapping quality, which is the result of **(C)** biases in read length. Consequently shorter reads are less likely to map, and to have lower mapping quality resulting in a systematic bias in read coverage over these regions of the mitochondrial genome. It appears that reads are more likely to prematurely truncate in regions of extreme (>60% A+T) content, but neither this nor homopolymer runs fully explain the pattern of read lengths and there was no evidence of site specific truncation of reads. Moreover, the short reads are not attributable to more closely spaced ribonucleotides, as sequences from the adaptor furthest from the sequencing primer were less not more prevalent among the short reads, supporting the conclusion that read truncation, rather than fragment size distribution, explains the regional variation. In Panel B and C, blue denotes reads mapped to the forward (light) and salmon the reverse (heavy) strand. Merge of blue and salmon show as purple.

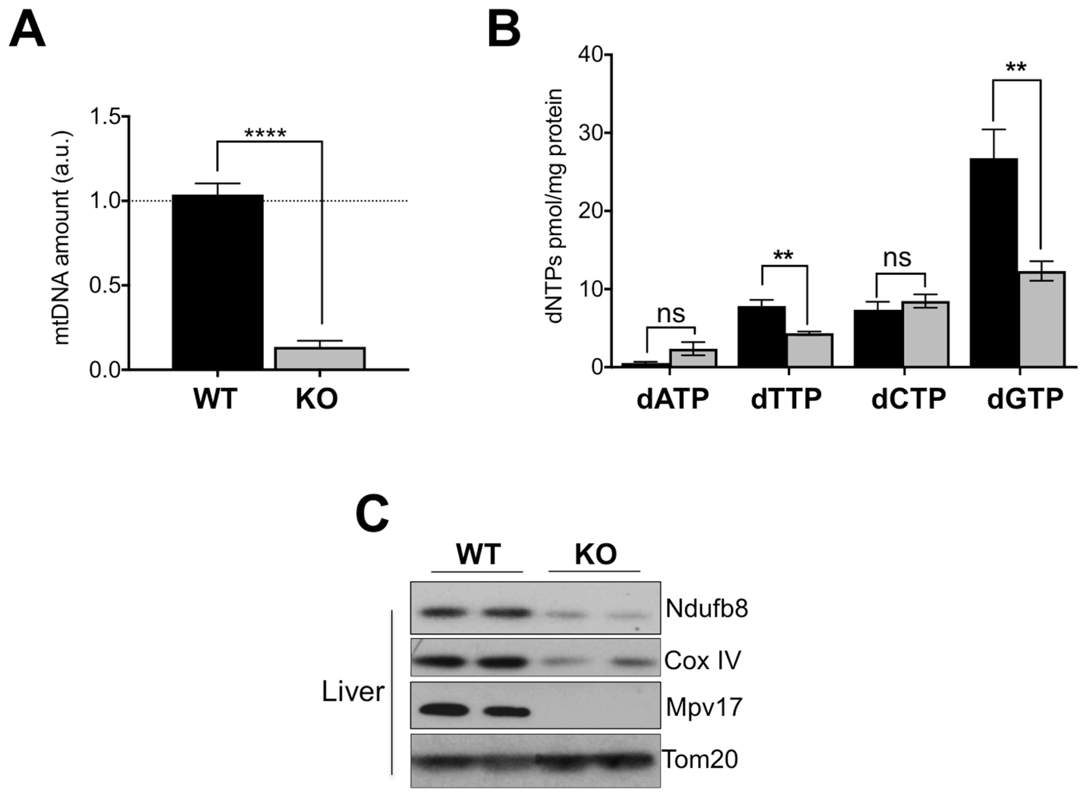

Figure S6. Livers of C57/B6 mice lacking the Mpv17 gene display the same mtDNA copy number, dNTP and OXPHOS defects as the CFW mouse studied previously. A) mtDNA copy number measured by qPCR; Data are expressed as mean ± SEM of n = 6. (T-test, *** P<0.001) **B**) dNTP pool sizes in mitochondria; All mice (n = 6) were sacrificed between 8 and 10 weeks of age. P values were obtained

using Mann-Whitney test (* $P<0.05$, ** $P<0.01$, NS, not significant $P>0.05$). and **C**) OXPHOS levels determined by immunoblotting, in liver of WT and Mpv17 KO mice, aged 2 months .

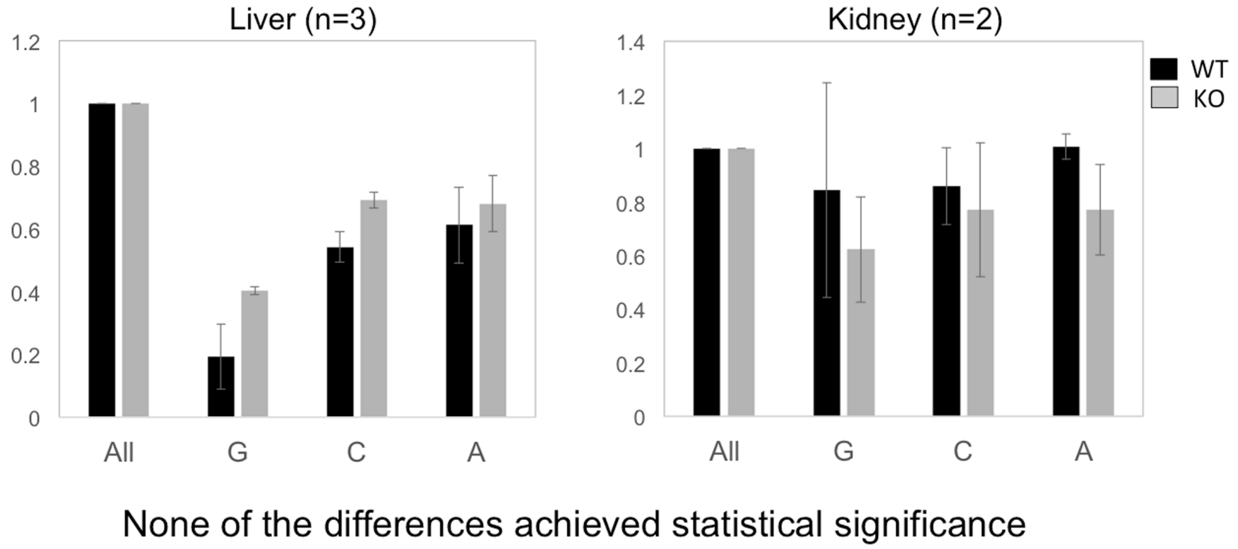

Figure S7. RNA synthesis driven by NTP extracts of wild-type and Mpv17 knockout mitochondria show no significant differences in an affected or an unaffected organ. Mitochondria were lysed and nucleotides extracted with methanol. The extracts (from liver or kidney) were incubated with RNA polymerase, template and ^{32}P-UTP, and two other NTPs. In columns labeled C, G or A the reaction required the mitochondrial extract to provide the CTP, GTP or ATP, respectively, to support RNA synthesis. The reference reactions contained no extract and all 4 NTPs. Liver has low dGTP and dTTP and mtDNA depletion, whereas kidney has dNTPs and mtDNA levels in the control range.

KO

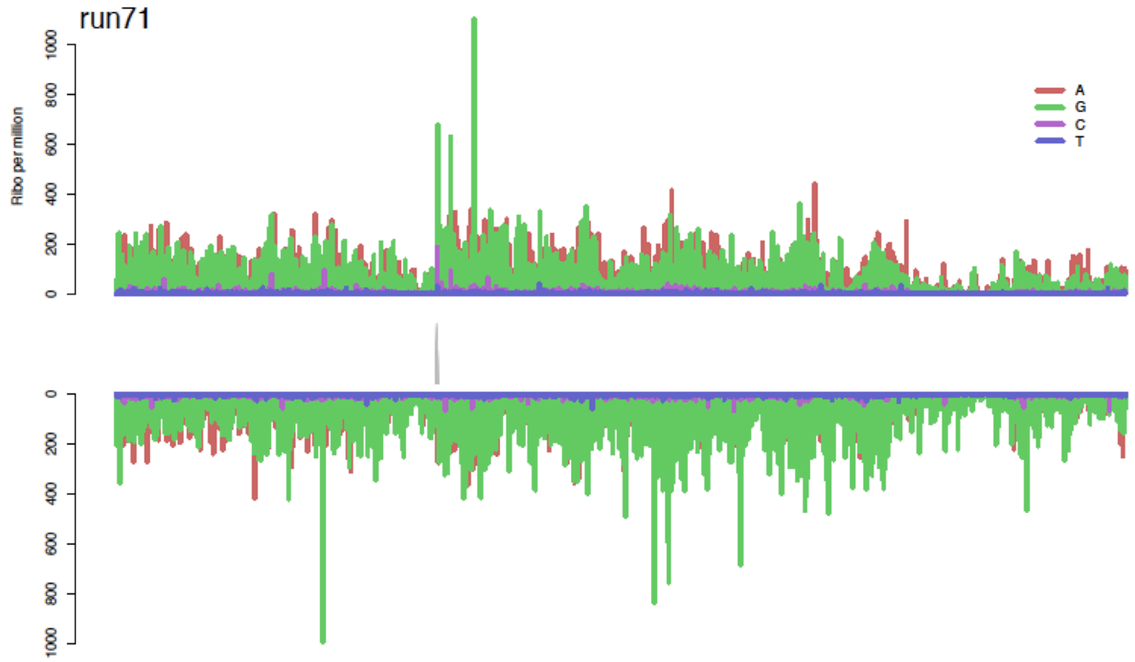

Figure S8. Enlarged image from Figure 3C. The distribution of the rNMPs in murine liver mtDNA lacking the Mpv17 gene (KO). The circular mitochondrial genome is displayed linearly with the first nucleotide far left and np 16,299 to the far right. Upper plots are H-strand, lower plots L-strand. The total of the all histogram bars over both strands for the WT plot sum to 1 million. The same is true for the KO plot.

Sample	Total bases	ML (all bases)	A	C	G	T	A	C	G	T
CM1	6.61E+08	1.26E-03	3.82E-04	3.43E-04	2.05E-04	3.25E-04	30.32%	27.22%	16.27%	25.79%
CM2	1.51E+08	5.51E-03	1.68E-03	1.51E-03	8.98E-04	1.43E-03	30.49%	27.40%	16.30%	25.95%
CM3	3.19E+08	2.60E-03	7.91E-04	7.12E-04	4.24E-04	6.74E-04	30.42%	27.38%	16.31%	25.92%
CM4	3.76E+08	2.21E-03	6.71E-04	6.04E-04	3.60E-04	5.72E-04	30.36%	27.33%	16.29%	25.88%
CM5	1.02E+08	8.16E-03	2.48E-03	2.23E-03	1.33E-03	2.11E-03	30.39%	27.33%	16.30%	25.86%
CM6	2.73E+08	3.04E-03	9.26E-04	8.33E-04	4.96E-04	7.88E-04	30.46%	27.40%	16.32%	25.92%

Table S1. Mpv17 deficiency does not alter the mutant load of brain mtDNA. Conventional next generation sequencing (that does not report rNMPs) was applied to libraries prepared from three control (CM1-3) and three Mpv17$^{-/-}$ mice (CM4-6) of 3 months of age. There was no significant difference in bases differing from the reference sequencing between the two groups of mice. Hence, the only evident abnormality of brain mtDNA in mice of this age is increased rGMP, see main text. Results calculated as previously described (10). ML= total number of misincorporated bases/total coverage. Misincorporation of bases is consistent across all samples.

References

1. Ding, J., Taylor, M.S., Jackson, A.P. and Reijns, M.A. (2015) Genome-wide mapping of embedded ribonucleotides and other noncanonical nucleotides using emRiboSeq and EndoSeq. *Nat Protoc*, **10**, 1433-1444.
2. Brennicke, A. and Clayton, D.A. (1981) Nucleotide assignment of alkali-sensitive sites in mouse mitochondrial DNA. *J Biol Chem*, **256**, 10613-10617.
3. Holmes, J.B., Akman, G., Wood, S.R., Sakhuja, K., Cerritelli, S.M., Moss, C., Bowmaker, M.R., Jacobs, H.T., Crouch, R.J. and Holt, I.J. (2015) Primer retention owing to the absence of RNase H1 is catastrophic for mitochondrial DNA replication. *Proc Natl Acad Sci U S A*, **112**, 9334-9339.
4. Jinks-Robertson, S. and Klein, H.L. (2015) Ribonucleotides in DNA: hidden in plain sight. *Nat Struct Mol Biol*, **22**, 176-178.
5. Keszthelyi, A., Daigaku, Y., Ptasinska, K., Miyabe, I. and Carr, A.M. (2015) Mapping ribonucleotides in genomic DNA and exploring replication dynamics by polymerase usage sequencing (Pu-seq). *Nat Protoc*, **10**, 1786-1801.
6. Wong-Staal, F., Mendelsohn, J. and Goulian, M. (1973) Ribonucleotides in closed circular mitochondrial DNA from HeLa cells. *Biochem Biophys Res Commun*, **53**, 140-148.
7. Martens, P.A. and Clayton, D.A. (1979) Mechanism of mitochondrial DNA replication in mouse L-cells: localization and sequence of the light-strand origin of replication. *J Mol Biol*, **135**, 327-351.
8. Reijns, M.A., Rabe, B., Rigby, R.E., Mill, P., Astell, K.R., Lettice, L.A., Boyle, S., Leitch, A., Keighren, M., Kilanowski, F. *et al.* (2012) Enzymatic removal of ribonucleotides from DNA is essential for mammalian genome integrity and development. *Cell*, **149**, 1008-1022.
9. Kasiviswanathan, R. and Copeland, W.C. (2011) Ribonucleotide discrimination and reverse transcription by the human mitochondrial DNA polymerase. *J Biol Chem*, **286**, 31490-31500.
10. Dalla Rosa, I., Camara, Y., Durigon, R., Moss, C.F., Vidoni, S., Akman, G., Hunt, L., Johnson, M.A., Grocott, S., Wang, L. *et al.* (2016) MPV17 Loss Causes Deoxynucleotide Insufficiency and Slow DNA Replication in Mitochondria. *PLoS Genet*, **12**, e1005779.
